# Increased mortality in CD43-deficient mice during sepsis

**DOI:** 10.1371/journal.pone.0202656

**Published:** 2018-09-18

**Authors:** Katherine T. Fay, Deena B. Chihade, Ching-Wen Chen, Nathan J. Klingensmith, John D. Lyons, Kimberly Ramonell, Zhe Liang, Craig M. Coopersmith, Mandy L. Ford

**Affiliations:** 1 Department of Surgery, Emory University School of Medicine, Atlanta, GA, United States of America; 2 Emory Critical Care Center, Emory University School of Medicine, Atlanta, GA, United States of America; 3 Emory Transplant Center, Emory University School of Medicine, Atlanta, GA, United States of America; Purdue University, UNITED STATES

## Abstract

CD43 is a large transmembrane protein involved in T cell activation. Previous studies of CD43^-/-^ mice in viral models have demonstrated a role for CD43 in Th1/Th2 skewing, activation of Foxp3+ Treg, and T cell apoptosis. However, the role of CD43 during sepsis has never been tested. Thus, we interrogated the role of CD43 during sepsis using a murine cecal ligation and puncture (CLP) model, and found that CD43^-/-^ mice demonstrated significantly worsened mortality compared to B6 mice following CLP. Phenotypic analysis of splenocytes isolated 24 h after septic insult revealed significantly increased apoptosis of central memory cells in both CD4^+^ and CD8^+^ T cell compartments in CD43^-/-^ septic mice compared to WT septic mice. Furthermore, CD43^-/-^septic mice exhibited a prominent Th2 skewing following sepsis relative to WT septic mice, as evidenced by a significant decrease in the frequency of IL-2^+^ CXCR3^+^ T_H_1 cells as a significant increase in the frequency of IL-4^+^ CCR4^+^ T_H_2 cells. Finally, septic CD43^-/-^ animals contained significantly fewer CD25^+^ Foxp3^+^ T_Reg_ cells as compared to WT septic animals. Importantly, depleting CD25^+^ Treg eliminated the increased mortality observed in CD43^-/-^ mice. Taken together, these data demonstrate an important role of CD43 in modulating immune dysregulation and mortality following sepsis.

## Introduction

Sepsis is defined as a life-threatening organ dysfunction caused by a dysregulated host response to infection [1]. Despite an annual incidence of up to 3 millions cases in the U.S. and a reported 42.5% mortality in severe cases [2], effective therapy once antibiotics and supportive care fails is still lacking. Once thought to be predominantly a disorder of excessive inflammation, the immunological derangements found in sepsis pathology are now understood to shift from a hyper-inflammatory phase to one of persistent, long-term immunosuppression [3–5]. For example, aberrant T cell activation [6, 7], upregulation of inhibitory proteins [8, 9], and extensive lymphocyte apoptosis [10] in the setting of high antigen exposure due to ineffective infection control result in host immune incompetence. These alterations in immunity in the later phase of sepsis place the patient at risk for opportunistic pathogens and secondary infections, resulting in increased long term morbidity and mortality. Some studies have shown that as many as 60% of patients succumb to these secondary infections during these later phases of sepsis [11]. Characterizing the immune incompetence of sepsis is imperative to further defining this disease process as well as allowing for development of possible immunomodulatory therapies.

Extensive lymphocyte apoptosis is a well-known feature of sepsis. Several postmortem studies have confirmed widespread apoptosis of lymphocytes most commonly seen in lymphoid organs, such as the spleen, as well as gastrointestinal lymphoid associated tissue (GALT) [10, 12]. The mechanism for apoptosis initiation seems to be multifactorial as multiple cell death pathways are activated in sepsis, including both extrinsic and intrinsic pathways [13]. Significant cellular depletion occurs across a multitude of lineages within both the adaptive and the innate immune systems, notably in CD4^+^ and CD8^+^ T cells, B cells, and dendritic cells [14–16]. This resultant lymphopenia significantly alters host response to subsequent infection, which often manifests as reactivation of latent viral infections such as CMV and EBV [17].

Not only is there a significant decrease in the number of circulating lymphocytes during sepsis, but the functionality of the remaining T cells is also dramatically altered. In particular, the balance of T helper cell populations, specifically T_H_1, T_H_2, T regulatory cells (T_reg_ cells), and T_H_17, is modified during sepsis. The axis of T_H_1/ T_H_2 cells, the two major subtypes of effector T cells, is implicated in a multitude of disease pathways. T_H_1 cells are a subset of T helper cells most commonly implicated in clearance of intracellular bacteria and cell-mediated immunity, while T_H_2 cells are associated with the humoral immune system and providing help for antibody production [18]. Evaluation of circulating lymphocytes in septic patients has shown an imbalance of T_H_1/ T_H_2 effector cells towards a T_H_2 skew [19], as well as decreasing frequency of T_H_1 cells associated with increased sepsis severity [20, 21]. Further, expansion of T_Reg_ cells was associated with T cell anergy in human septic patients [22], but more mechanistic studies using both loss- and gain-of-function approaches in murine models have revealed that Foxp3^+^ Treg are likely beneficial in the setting of sepsis [23, 24]. Additionally, blockade of IL-17A results in improved survival, suggesting that Th17 cells may negatively contribute to sepsis mortality [25].

Glycosylation of T cell surface receptors also plays an important role in altering activation and function in the inflammatory setting. CD43 is a large, highly glycosylated transmembrane protein, abundant on T cells, and has been implicated in several lymphocytic processes. Blockade of CD43 resulted in decreased trafficking of lymphocytes to lymphoid tissues, implicating a role for CD43 in T cell trafficking both at baseline and at sites of inflammation [26, 27]. Additionally, CD43 has been associated with T helper cell differentiation. Ex-vivo stimulation of CD43^-/-^ lymphocytes showed that these cells produce significantly more IL-4 compared to WT cells, suggesting they are skewed towards a Th2 phenotype [28]. CD43 also plays a role in cell death in that upregulation of CD43 on the T cell surface is associated with T cell exhaustion [3] and increased enhanced apoptosis via binding of galectin-1[29]. Finally, CD43 is expressed on Foxp3^+^ Treg and is upregulated during Treg activation. Further, one study showed that sialoadhesin (Sn) expressed by monocytic cells can bind to CD43 expressed on Foxp3^+^ Treg and negatively regulate their expansion [30].

Despite this understanding of the role of CD43 in T cell activation and regulation, very little is known about the role of CD43 in sepsis. Delayed upregulation of CD43 has been implicated as a possible mechanism for the immune dysfunction observed in septic mice exposed to alcohol chronically [31]; however, beyond this, limited data exists. Here we sought to further characterize the role of CD43 in sepsis given its association with several T cell functions that are dysregulated in sepsis.

## Methods

### Animals

Six week old C57/B6 (wild type or WT) mice of both male and female gender were acquired from Jackson Laboratory (Bar Harbor, ME) and allowed to acclimate for one week prior to utilization in experiments. A breeder pair of CD43^-/-^ mice was also acquired from Jackson Laboratory and a CD43^-/-^ colony was established within our animal facility at Emory University. All mice were housed in the same animal facility at Emory University and had free access to chow and water. All experiments were performed in accordance with the National Institutes of Health Guidelines for the Use of Laboratory Animals and under approval of the Institutional Animal Care and Use Committee at Emory University School of Medicine (protocol DAR 2002473-101819N).

### Sepsis model

CD43^-/-^ and B6 male and female mice were subjected to cecal ligation and puncture as previously published [32]. No differences in CLP survival were observed between male and female mice in these experiments (data not shown). Mice were anesthetized with isoflurane and a small midline incision was made. The cecum was externalized and ligated 1cm from its base utilizing 4–0 silk suture. The cecum was then punctured twice with a 25-gauge needle, a small amount of stool was extruded into the abdominal cavity, and the intestine was placed back within the abdomen. The midline peritoneal incision was closed with 4–0 silk suture and the skin incision with animal surgical adhesive. Sham control animals underwent the same procedure without ligation and puncture of the cecum. All animals received buprenorphine (0.1mg/kg) preoperatively for pain relief and 1mL of normal saline for intraoperative fluid losses as well as antibiotics (ceftriaxone 25mg/kg and metronidazole 12.5mg/kg) subcutaneously postoperatively. Antibiotics were continued on a q12hr dosing schedule for 48 hours postoperatively. Where indicated, animals were sacrificed at 24 h for analysis of immune cells.

### Phenotypic flow cytometric analysis

For phenotypic analysis, animals were sacrificed at 24hrs utilizing CO_2_ euthanasia and their spleens were harvested. The spleens were processed into single-cell suspensions and the number of cells per mL of suspension was obtained via a Nexcelom Auto Cellometer. 2x10^6^ cells were plated into a 96-well plate and stained for extracellular markers CD4—Pacific Blue (BD Biosciences, clone RM4-5), CD8—Pacific Orange (Invitrogen, clone MCD0830), CD3—APCCy7 (BioLegend, clone 17A2), CD44—PerCP (BioLegend, IM7), CD62L—APC (eBioscience, clone MEL-14), CD43—FITC (BioLegend, clone 1B11), and CD43—PE (BD Pharmingen, S7). TruCount Beads from BD Pharmingen were prepared according to the manufacturer’s instructions and used to determine absolute cell counts.

### Intracellular cytokine staining

For cytokine analysis, 2 x 10^6^ splenic cells were plated into a 96-well plate. Cells were suspended in RMPI 1640 culture medium and incubated for four hours utilizing phorbol 12-myristate 13-acetate (30ng/mL) and ionomycin (400ng/mL) with 10μg/mL of Brefeldin A at 37°C. After stimulation, cells were then stained for the following intracellular and extracellular markers CD4—Pacific Blue (BD Biosciences, clone RM4-5), CD8—Pacific Orange (Invitrogen, clone MCD0830), CD25—FITC (BioLegend, clone PC61), CD3—Alexa 700 (BD Biosciences, 500A2), FOXP3—APC (eBioscience, FJK-16S), IL-2—FITC (BD Pharmingen, clone JES6-5H4), IL-4—PE (BioLegend, clone 11B11), CCR4—PE Cy7 (BioLegend, clone 2G12), CXCR3—APC (BioLegend, clone CXCR3-173), and IL-17A—PE (eBioscience, clone eBio17B7). Samples were run on an LSR II flow cytometer (BD Biosciences) and subsequent data was analyzed with FlowJo 10.0.8rl software (Tree Star, San Carlos, CA).

### Apoptosis quantification

To determine the frequency of lymphocytic populations, 2 x 10^6^ splenic cells were plated into a 96-well plate and were processed utilizing a commercially available 7-AAD and Annexin V kit (BioLegend) following the manufacturer’s instructions. Cells were stained extracellularly with anti-CD4, anti-CD8, anti-CD44, and anti-CD62L (all BD Pharmingen) to identify cell populations of interest. Apoptotic cells were identified as being both 7-AAD^+^ and Annexin V^+^.

### In vitro Th1 polarization and adoptive Th1 transfer in CD43^-/-^ septic mice

Spleens from female CD43^-/-^ mice were processed to a single-cell suspension into MACS buffer. CD4+ T cells were harvested and purified using the CD4+ T cell isolation Kit II (Miltenyi Biotec #130-104-454) adhering to the manufacturer’s protocol using LS magnetic column separators (130-042-401) and autoMACS Running Buffer. Cells were counted for confluency and treated with Biotin-labeled antibody cocktail containing CD8a, CD11b, CD11c, CD19, CD45R B220 isotype, CD49b DX5 isotype, CD105, anti-MHC Class II, Ter-119 and TCR gamma/delta followed by microbead-conjugated secondary antibody for negative selection. After incubation, the suspension was run through magnetic columns and CD4+ flow-through was collected in R10 media (RPMI medium 1640 with 50 μM 2-mercaptoethanol and 2 mM glutamine, supplemented with 10% FCS). CD4^+^ cells were plated onto anti-CD3 (BDPharm, cat # 550277) and anti-CD28 (BioXcell clone 37.51, cat# 102101) coated wells and incubated in the presence of 10 ug/ml anti-IL-4 capture antibody, 10 ng/ml recombinant IL-12, and 5 ng/ml recombinant IL-2 for 72 hours. On day of transfer, cells were collected and measured at >85% confluency. Cells were resuspended in sterile PBS at 5 x 10^6^ cells per 100 ul PBS. CD43^-/-^ male and female mice underwent Th1 adoptive transfer through tail vein injection of 5 x 10^6^ treated cells in PBS versus PBS vehicle alone on post-operative day one status post CLP induced sepsis. Mice were monitored for survival.

### In vivo cytokine blockade and Treg depletion

For IL-4 and IL-17 neutralization studies, anti-IL-4 (clone 11B11, 200 ug/dose) or anti-IL-17A (clone 17F3, 250 ug/dose) (both BioXCell) were administered to CD43^-/-^ hosts on days 0, 2, 4, and 6 post-CLP. For Treg depletion, CD43^-/-^ mice and their C57/B6 counterparts were injected i.p. with 200 ug PC61 anti-CD25 mAb (BioXCell) 24 hours prior to CLP. Mice underwent CLP and were injected with 200 ug of PC61 post-operative day 3 and monitored for survival for 7 days.

### Statistical analysis

Statistical analyses were performed utilizing GraphPad Prism 6.0 software (San Diego, CA) and are presented as mean ± SEM. Data were tested for Gaussian distribution using the D’Agostino-Pearson omnibus normality test. For multiple group comparisons, one-way ANOVA followed by Tukey post-test or non-parametric Kruskall-Wallis test followed by Dunn’s test were utilized. Survival curves were compared by log-rank test. A p value of <0.05 was considered statistically significant.

## Results

### CD43 (isoform 1B11) is significantly upregulated on CD4^+^ and CD8^+^ T cells during sepsis

**Fig 1:**
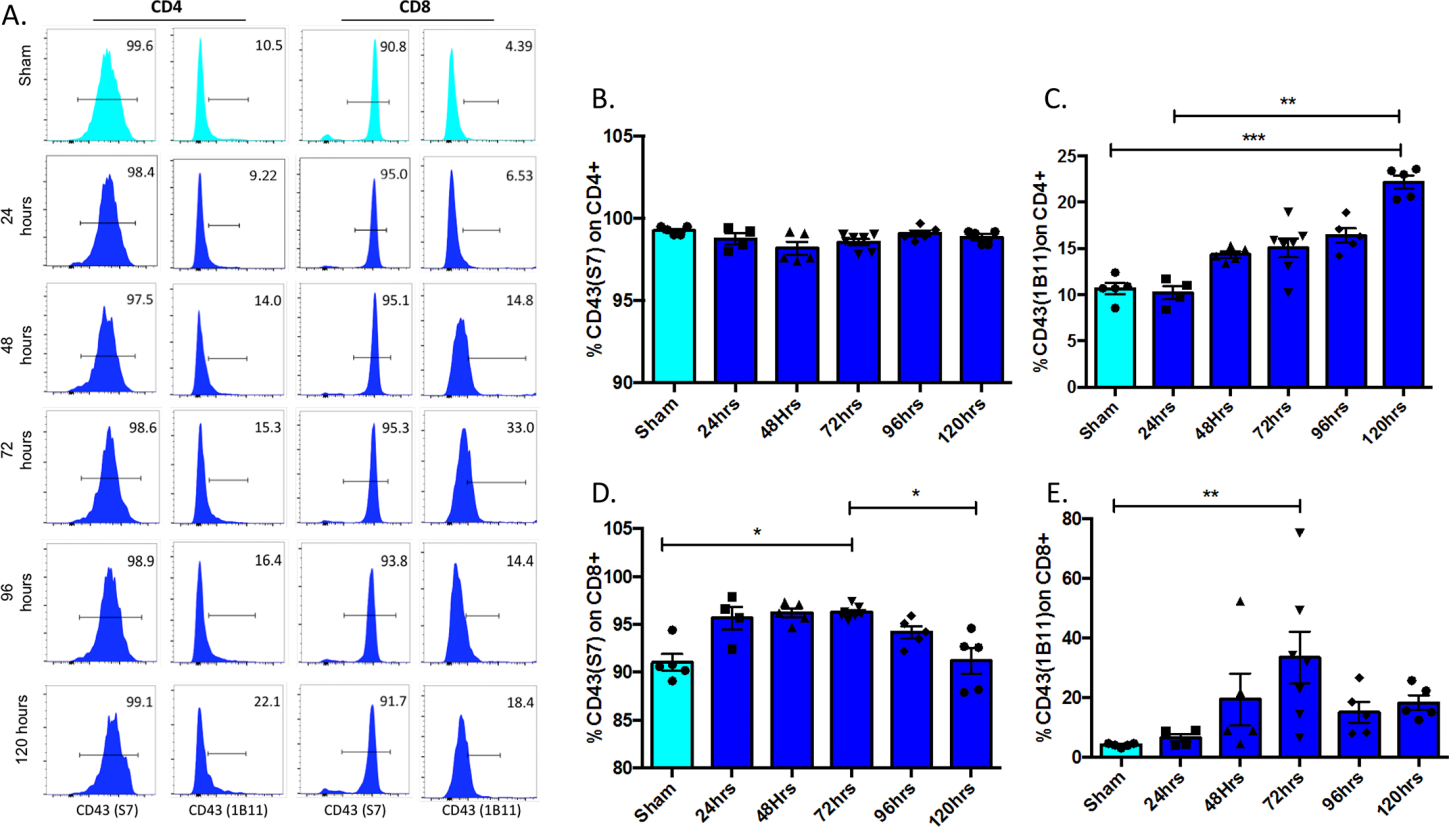
Frequencies of 1B11^+^ CD4^+^ and CD8^+^ T cells increase during sepsis. B6 mice were subjected to CLP and sacrificed at the indicated timepoints. Spleens were harvested and CD4^+^ and CD8^+^ T cells were assessed for staining with the S7 or 1B11 clones that recognize distinct glycoforms of CD43. A) Representative histograms of S7 and 1B11 staining on CD4^+^ and CD8^+^ splenic T cells. B) Summary data of S7 staining on CD4^+^ T cells harvested from spleens at indicated time points post-CLP or from sham mice revealed no significant changes over time. C) Summary data of 1B11 staining on CD4^+^ T cells revealed a significant increase from sham mice to septic mice at 120hrs (10.7±0.6 vs 22.2±0.7, p = 0.0009) as well as a significant increase between septic mice at 24hrs and 120hrs (10.2±0.7 vs 22.2±0.7, p = 0.002). D) Summary data of S7 staining on CD8^+^ T cells revealed a significant increase from sham to sepsis at 72hrs (91±0.9 vs 96.3±0.2, p = 0.02) that then decreased again at 120hrs (96.3±0.2 vs 91.2±1.3, p = 0.03). E) Summary data of 1B11 staining on CD8^+^ T cells revealed an increase from sham to sepsis at 72hrs (4.1±0.3 vs. 18.3±2.5 vs p = 0.004). Data shown are 4–6 mice per group, and are representative of 3 independent experiments. Statistical comparisons were performed by one-way ANOVA followed by Kruskall-Wallis non-parametric post-tests.

The kinetics of CD43 expression was evaluated over a 120 hr time course after sepsis induction. Differential glycosylation of CD43 results in the expression of two common glycoforms, recognized by clones S7 and 1B11. The glycoform recognized by S7 is constitutively expressed on both CD4^+^ and CD8^+^ T cells, while the glycoform recognized by 1B11 is upregulated following T cell activation and returns to baseline on quiescent memory T cells. In line with these previously published results, in that ~100% of splenic CD4^+^ T cells and ~90% of splenic CD8^+^ T cells expressed the antigen S7 in sham animals, while ~10% of CD4^+^ and 5% of CD8^+^ cells expressed the 1B11 antigen in sham controls (Fig 1A and 1B, light blue bars). Following sepsis induction, the frequency of S7^+^ CD4^+^ T cells did not change significantly over the course of 96 hr (Fig 1A and 1B). However, the frequency of the 1B11^+^ CD4^+^ T cells was significantly increased at 120 hr post sepsis (Fig 1A and 1C). The frequency of S7^+^ CD8^+^ T cells increased significantly from sham controls at 72 hr post sepsis (Fig 1A and 1D), and more strikingly, the frequency of 1B11^+^ CD8^+^ T cells also increased from ~5% in sham animals to ~30% at 72 hr (Fig 1A and 1E). The frequency of 1B11^+^ of CD8^+^ T cells returned to baseline levels at 96 and 120 hours post-CLP.

### CD43^-/-^ mice exhibit significantly worsened sepsis survival

**Fig 2:**
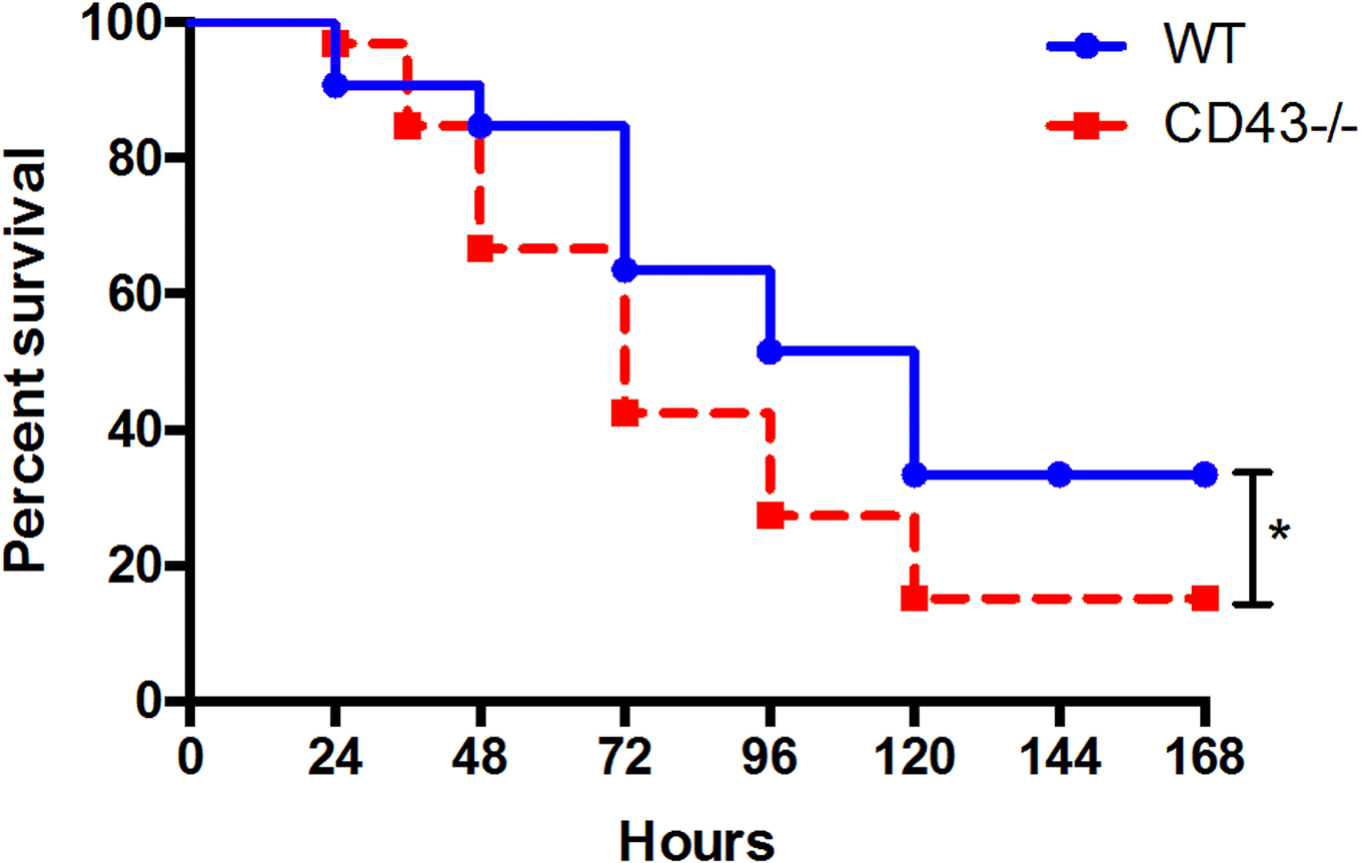
Loss of CD43 significantly worsens sepsis survival. B6 and CD43^-/-^ mice were subjected to CLP and monitored for survival. CD43^-/-^ mice exhibited significantly increased mortality compared to WT controls following CLP (33.3% vs 15.2%, p = 0.04, n = 34/ group). Mortality was compared by log-rank test.

Given the increase in frequency of cells expressing the CD43 isoform 1B11 in both CD4^+^ and CD8^+^ T cell populations following CLP we hypothesized that CD43 might be playing a pathophysiologic role during sepsis. To test this, CD43^-/-^ mice were utilized to assess the role of this T cell surface receptor in sepsis survival. After being followed for 7 days post CLP, CD43^-/-^ mice demonstrated significantly worsened survival compared to WT controls (Fig 2).

### Numbers of CD4^+^ and CD8^+^ cells in CD43^-/-^ septic vs. WT septic mice are not different

**Fig 3:**
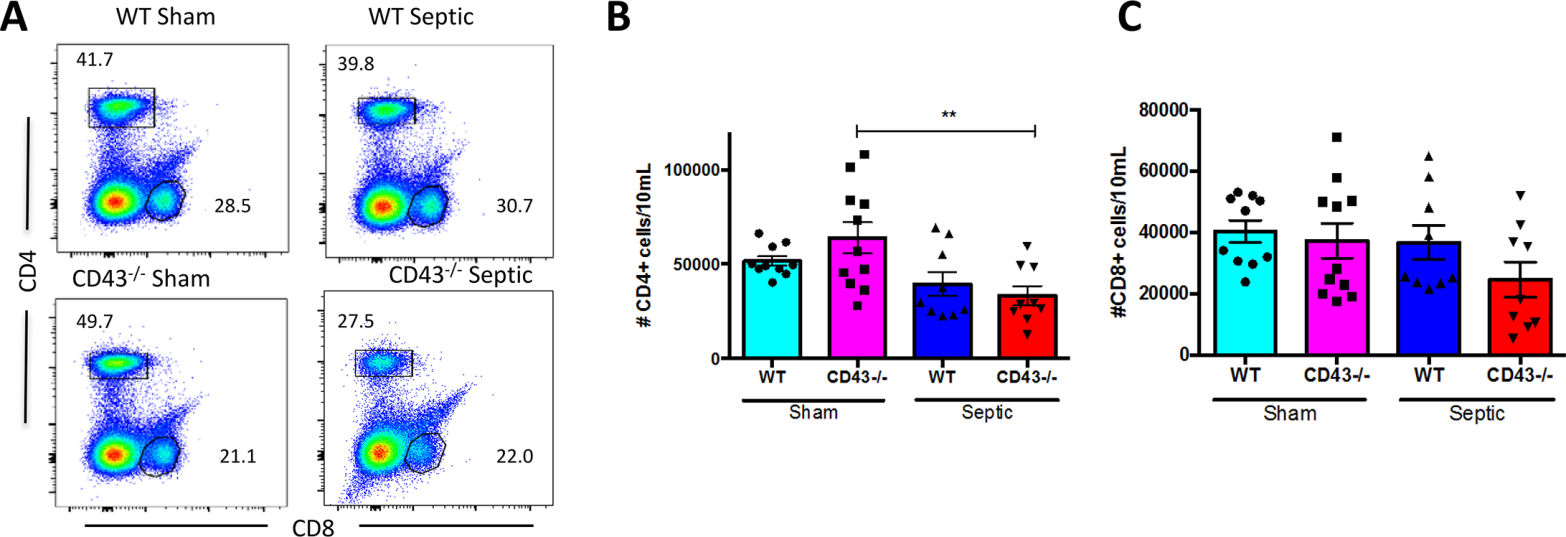
CD43 deficiency does alter numbers of CD4^+^ or CD8^+^ T cells during sepsis. B6 and CD43^-/-^ mice were subjected to CLP and spleens were harvested at 24h and analyzed by flow cytometry. A) Representative flow cytometry of frequencies of CD4^+^ and CD8^+^ T cells. B) Summary data of numbers of CD4^+^ T cells revealed a significant decrease in CD4^+^ T cell counts in septic CD43^-/-^ mice compared to sham CD43^-/-^ mice (33345±5137 vs. 63792±8225, p = 0.007). However, there were no differences between the sham and septic groups in WT mice (51629±2559 vs 39455±6336, p = 0.4), nor between the two septic groups (25918±1500 vs 23377±3698, p>0.9). C) Summary data of numbers of CD8^+^ T cells revealed no difference in CD8^+^ T cell counts between CD43^-/-^ sham and WT sham groups (37208±5632 vs 40318±3567, p = 0.95). Similarly, there was no difference in the number of CD8^+^ T cells between the septic groups (24609±5684 vs 36618±5539, p = 0.4). Data shown are cumulative from 2 separate experiments with a total of 9-11animals per group. Statistical comparisons were performed by one-way ANOVA followed by Kruskall-Wallis non-parametric post-tests.

Previous studies have shown the CD43 deficient mice have normal T lymphocytes development and have similar circulating T lymphocyte populations to wild type mice [33]. Similarly, analysis of the splenic compartment revealed no differences in the number of CD4^+^ T cells between CD43^-/-^ mice and WT mice either after sham laparotomy or CLP (Fig 3A and 3B). However, there was a sepsis-related decrease in the number of CD4^+^ T cells in CD43^-/-^ mice from sham controls and a trend towards a decrease in WT mice. Additionally, there were no alterations in the number of CD8^+^ T cells between the sham and septic groups, between CD43^-/-^ mice and WT mice (Fig 3A and 3C).

**Fig 4:**
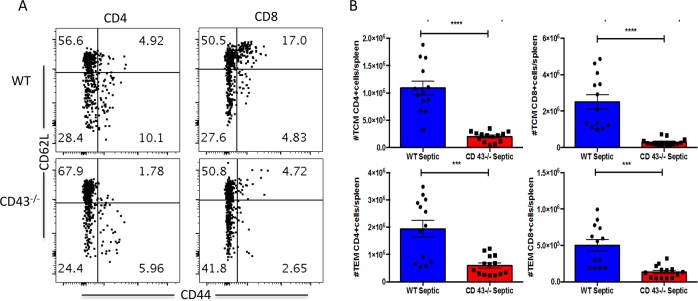
Absolute Numbers of CD4^+^ and CD8^+^ central and effector memory cells are decreased in CD43^-/-^ relative to WT septic animals. WT and CD43^-/-^ animals were subjected to CLP and spleens were harvested at 24h. There are significant decreases in both T_CM_ and T_EM_ compartments of CD4^+^ and CD8^+^ T cells between septic groups. (***p = 0.0001). Data shown are from 13–14 mice per group complied from three independent experiments. Statistical comparisons were performed by Mann-Whitney non-parametric test.

In examining memory T cell subsets, however, we identified significant differences between the groups (Fig 4A). Absolute numbers of CD4^+^ and CD8^+^ central memory (CD44^hi^ CD62L^hi^) (Fig 4B and 4C) and effector memory (CD44^hi^ CD62L^lo^) (Fig 4D and 4E) cells were statistically significantly decreased in CD43^-/-^ septic mice as compared to WT septic mice. Of note, absolute numbers of central and effector CD4 and CD8+ T cells were not different between WT and CD43^-/-^ sham controls (data not shown).

### CD43^-/-^ septic mice exhibit increased apoptosis of central memory T cells

**Fig 5:**
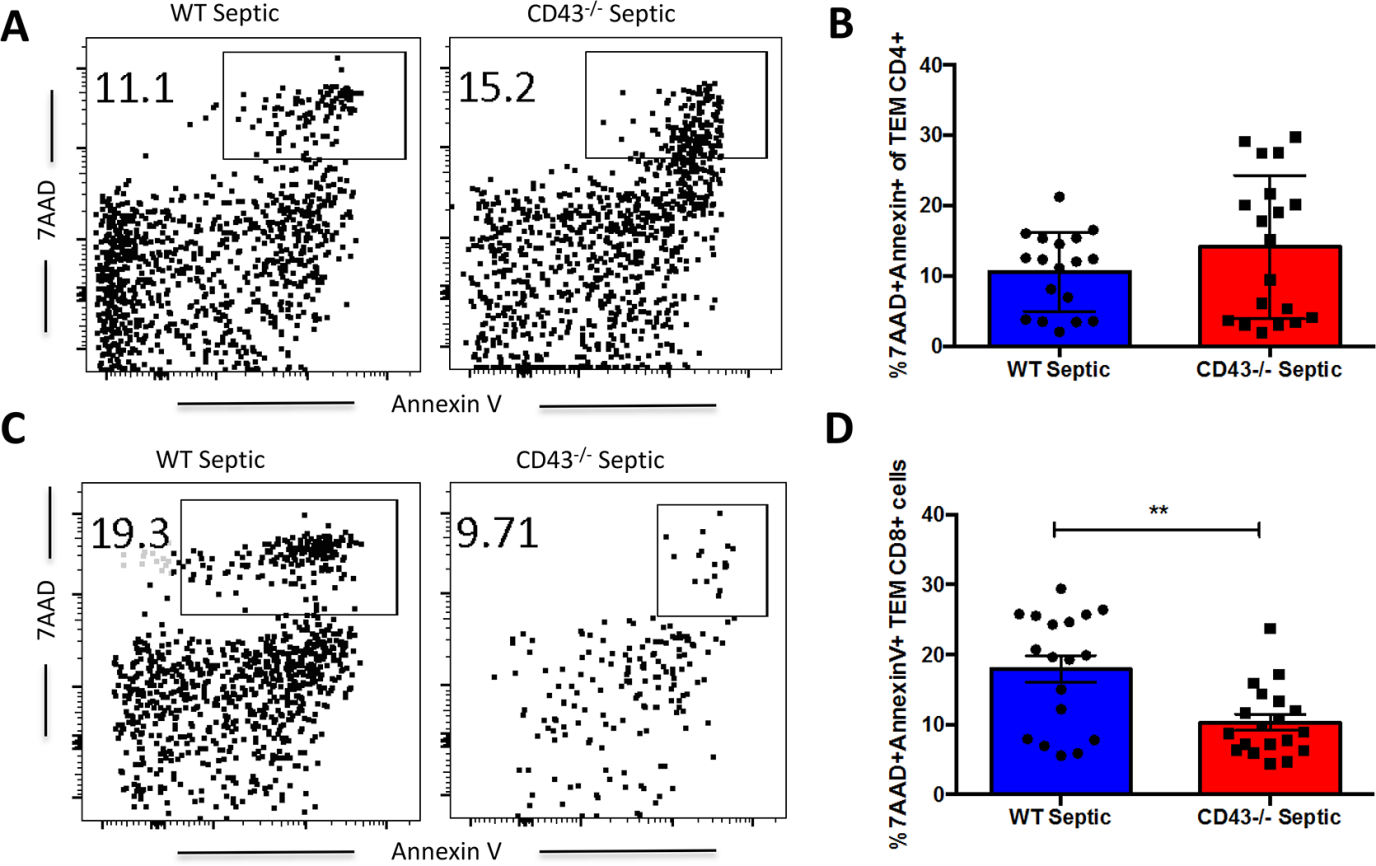
CD43^-/-^ septic mice exhibit decreased frequencies of apoptotic CD8^+^ T_EM_ as compared to WT septic mice. B6 and CD43^-/-^ mice were subjected to CLP and spleens were harvested at 24h and frequencies of apoptotic cells were assessed by 7-AAD and Annexin V staining. A, C) Representative 7-AAD and Annexin V staining of CD44^HI^ CD62L^LO^ CD4^+^ and CD8^+^ T_EM_. B) There was no difference in frequency of apoptosis in CD4^+^ effector memory cells, defined as 7-AAD^+^ Annexin V^+^ CD44^HI^ CD62L^LO^ CD4^+^ cells, in CD43^-/-^ septic mice compared to WT septic mice (14.1±2.3 vs 10.6±1.3, p = 0.4). D) There was a significant decrease in apoptosis of CD44^HI^ CD62L^LO^ CD8^+^ T_EM_ in CD43^-/-^ septic mice compared to WT septic mice (10.3±1.1 vs 17.9±1.9, p = 0.006). Data shown are cumulative from 3 independent experiments with a total of 18–20 animals per group. Statistical comparisons were performed by Mann-Whitney non-parametric test.

**Fig 6:**
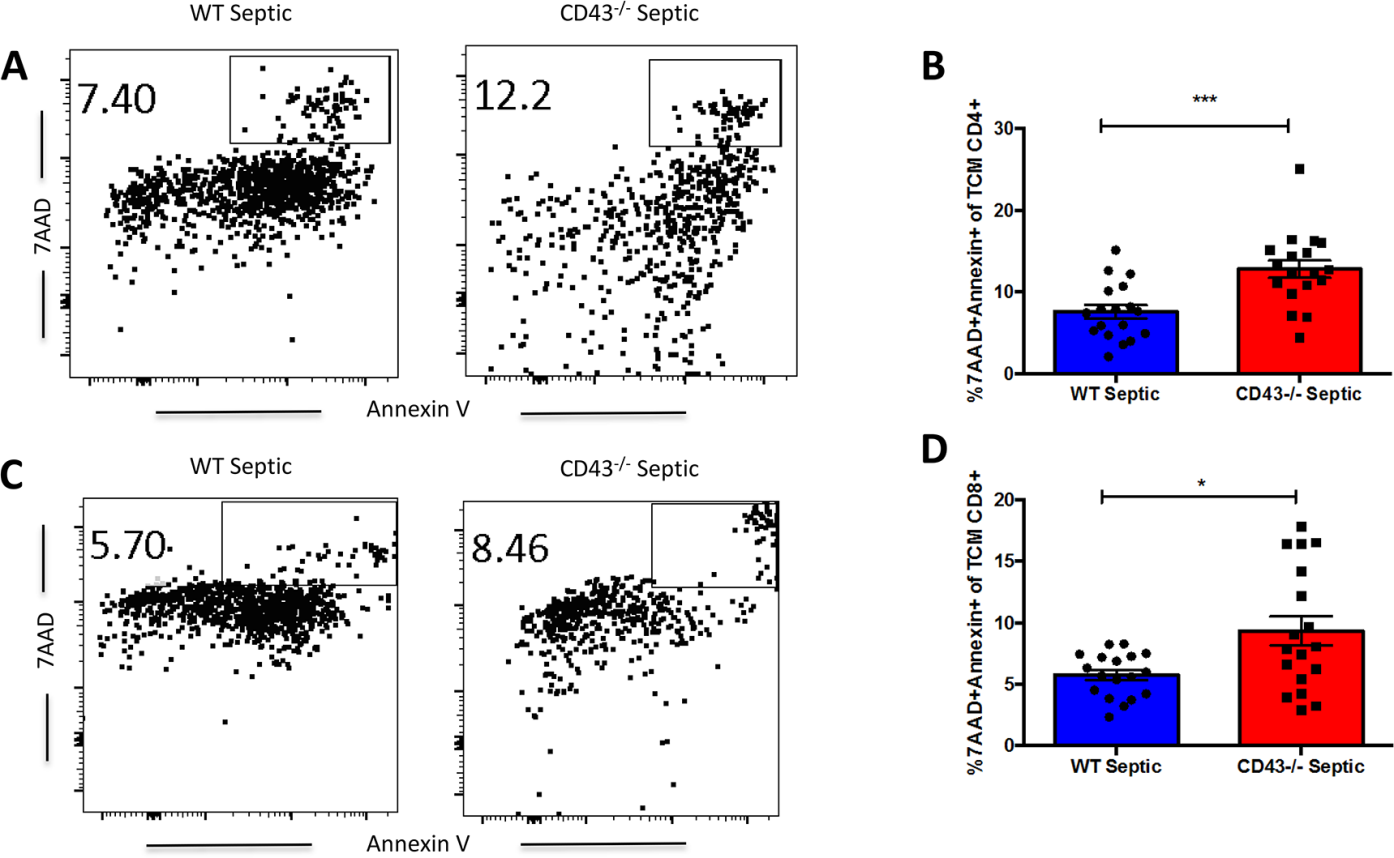
CD43^-/-^ septic mice exhibit increased frequencies of apoptotic CD4^+^ and CD8^+^ T_CM_ as compared to WT septic mice. B6 and CD43^-/-^ mice were subjected to CLP and spleens were harvested at 24h and frequencies of apoptotic cells were assessed by 7-AAD and Annexin V staining. A, C) Representative 7-AAD and Annexin V staining of CD44^HI^ CD62L^HI^ CD4^+^ (A) and CD8^+^ (C) T_CM_. B) There was a significant increase in frequency of apoptotic CD4^+^ T_CM_, defined as 7-AAD^+^ Annexin V^+^ CD44^HI^ CD62L^HI^ CD4^+^ cells, in CD43^-/-^ septic mice compared to WT septic mice (12.8±1.1 vs 7.6±0.8, p = 0.0005). D) There was a significant increase in frequency of apoptotic CD8^+^ T_CM_, defined as 7-AAD^+^ Annexin V^+^ CD44^HI^ CD62L^HI^ CD8^+^ cells, in CD43^-/-^ septic mice compared to WT septic mice (9.3±1.2 vs 5.8±0.4, p = 0.008). Data shown are cumulative from 3 independent experiments with a total of 18–20 animals per group. Statistical comparisons were performed by Mann-Whitney non-parametric test.

**Fig 7:**
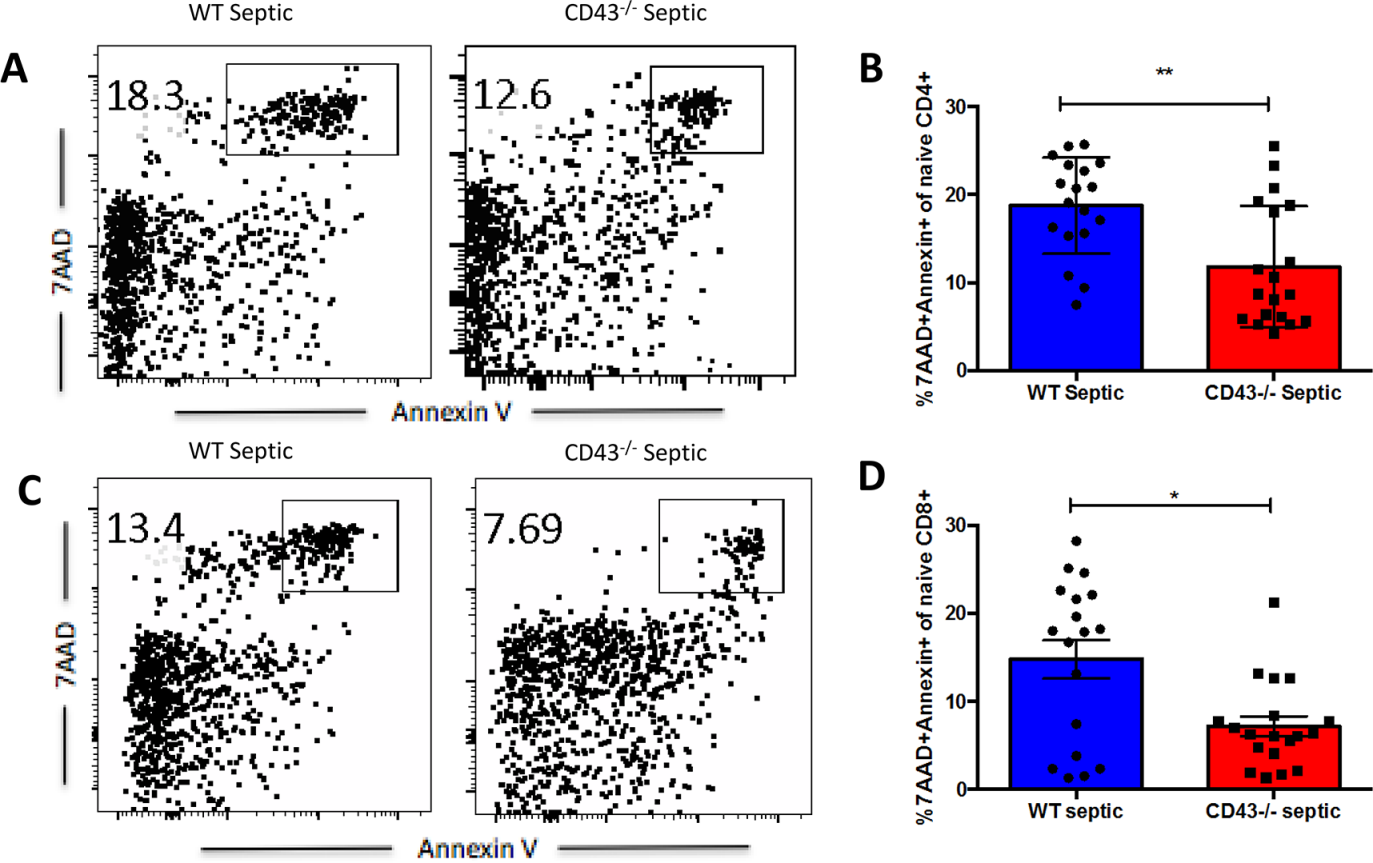
CD43^-/-^ septic mice exhibit decreased frequencies of apoptotic CD4^+^ and CD8^+^ naïve T cells as compared to WT septic mice. B6 and CD43^-/-^ mice were subjected to CLP and spleens were harvested at 24h and frequencies of apoptotic cells were assessed by 7-AAD and Annexin V staining. A, C) Representative 7-AAD and Annexin V staining of naïve CD44^LO^ CD62L^HI^ CD4^+^ and CD8^+^ T cells. B) CD43^-/-^ septic mice exhibited a decreased frequency of apoptotic naïve CD4^+^ T cells, defined as 7-AAD^+^ Annexin V^+^ CD44^LO^ CD62L^HI^ CD4^+^ cells, compared to WT septic mice (11.8±1.6 vs 18.8±1.3, p = 0.002. D) CD43^-/-^ mice exhibited a decreased frequency of apoptotic naïve CD4^+^ T cells, defined as 7-AAD^+^ Annexin V^+^ CD44^LO^ CD62L^HI^ CD8^+^ cells, compared to WT mice after sepsis induction (7.2±1.1 vs 14.8±2.2, p = 0.02). Data shown are cumulative from 3 independent experiments with a total of 18–20 animals per group. Statistical comparisons were performed by Mann-Whitney non-parametric test.

We next interrogated whether these differences in cell numbers in the CD43^-/-^ TCM and TEM compartments were attributable to alterations in T cell apoptosis during sepsis. Effector memory cells, defined as CD44^HI^ CD62L^LO^, within the CD4^+^ T cell compartment showed no differences in apoptosis between CD43^-/-^ mice compared to WT septic mice (Fig 5A and 5B). However, within the CD8^+^ T cell compartment, CD43^-/-^ mice have significantly decreased apoptosis of effector memory cells compared to WT septic mice (Fig 6C and 6D). Evaluation of central memory cell populations, defined as CD44^HI^ CD62L^HI^ cells, demonstrated a significant increase in apoptosis in CD43^-/-^ septic mice compared to WT septic mice in both CD4^+^ (Fig 7A and 7B) and CD8^+^ (Fig 7C and 7D) T cell populations. In contrast, in both CD4^+^ and CD8^+^ compartments, CD43^-/-^ septic mice exhibited significantly decreased frequencies of apoptotic naive cells compared to WT septic mice (Fig 7A and 7B). Taken together, these findings suggest a predominant loss of specifically central memory cells in CD43^-/-^ septic mice in both CD4^+^ and CD8^+^ T cell populations as a result of apoptosis.

### CD43^-/-^ septic animals exhibit a decrease in IL-2-secreting CD4^+^ T cells and an increase in IL-4-secreting CD4^+^ T cells relative to WT septic animals

**Fig 8:**
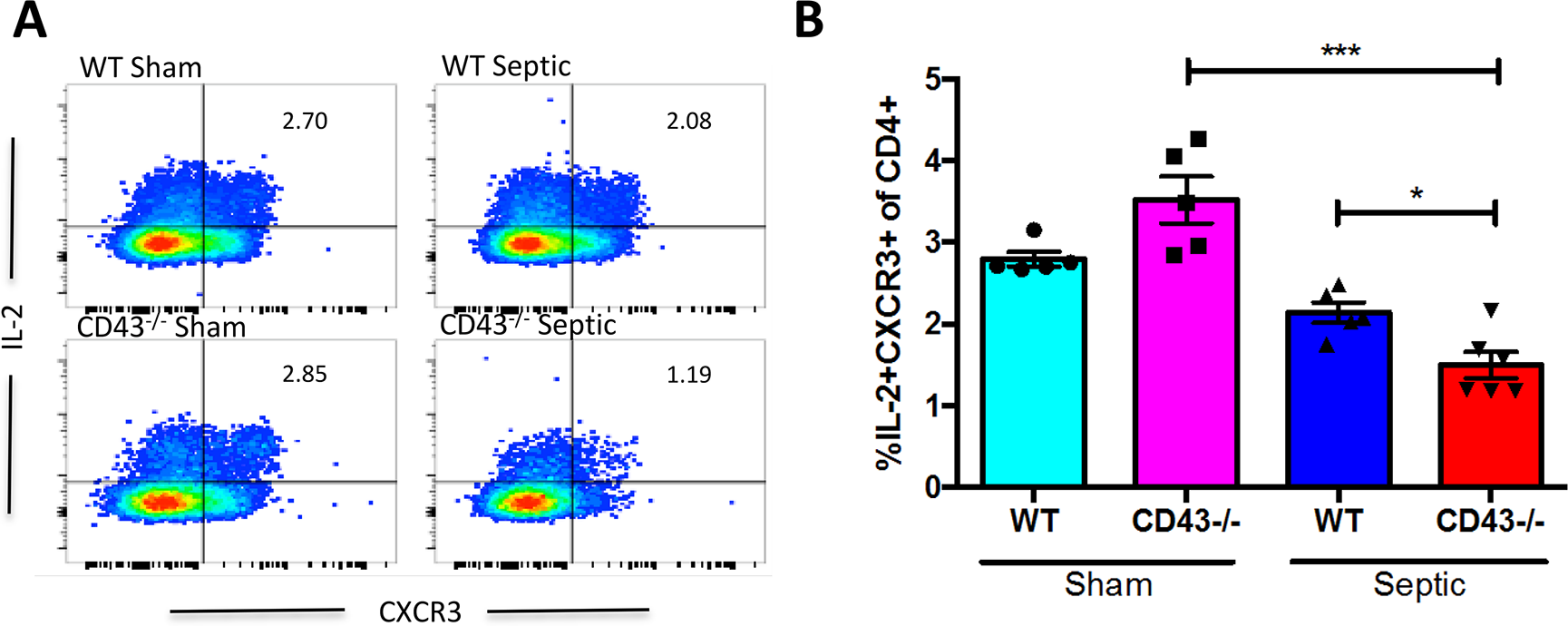
CD43^-/-^ mice exhibit reduced frequencies of CXCR3^+^ IL-2-secreting cells relative to WT mice following sepsis. B6 and CD43^-/-^ mice were subjected to CLP. Spleens were harvested at 24h and restimulated ex vivo with PMA/ionomycin. A) Representative flow cytometry depicting IL-2^+^ CXCR3^+^ CD4^+^ T cells within each group. B) Summary data showing no difference in the frequency of IL-2^+^ CXCR3^+^ CD4^+^ cells between CD43^-/-^ and WT sham groups (3.5±0.3 vs 2.8±0.1, p>0.9). Following CLP, CD43^-/-^ mice exhibited a significant reduction in the frequency of IL-2^+^ CXCR3^+^ CD4^+^ T cells as compared to WT controls (1.5±0.2 vs 2.1±0.1, p = 0.03). Data shown are 4–6 mice per group, and are representative of 3 independent experiments. Statistical comparisons were performed by one-way ANOVA followed by Kruskall-Wallis non-parametric post-tests.

**Fig 9:**
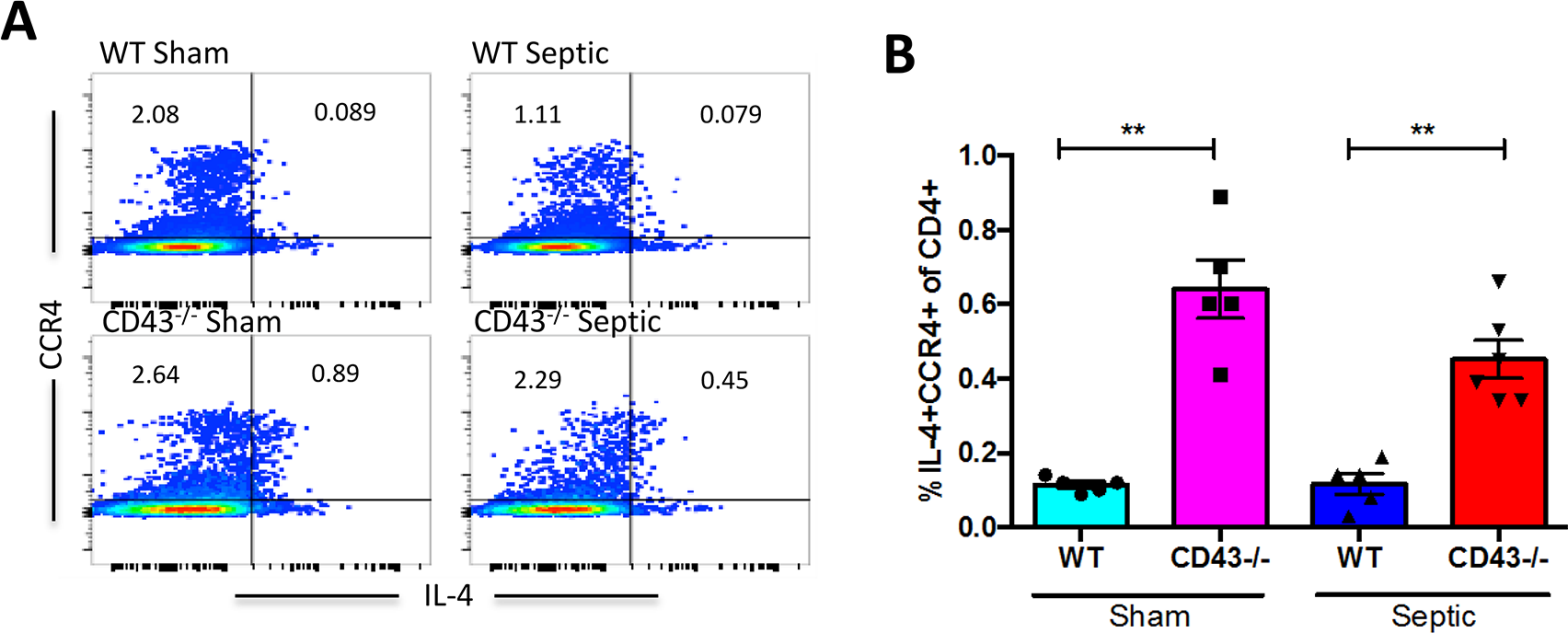
CD43^-/-^ mice exhibit increased frequencies of CCR4^+^ IL-4-secreting cells relative to WT mice following sepsis. B6 and CD43^-/-^ mice were subjected to CLP. Spleens were harvested at 24h and restimulated ex vivo with PMA/ionomycin. A) Representative flow cytometry depicting IL-4^+^ CCR4^+^ CD4^+^ T cells within each group. B) Summary data showing CD43^-/-^ mice exhibited a significant increase in IL-4^+^ CCR4^+^ CD4^+^ T cells compared to WT mice in both sham (0.6±0.1 vs 0.1±0.01, p = 0.007) and septic groups (0.5±0.05 vs 0.1±0.02, p = 0.002). Data shown are 4–6 mice per group, and are representative of 3 independent experiments. Statistical comparisons were performed by one-way ANOVA followed by Kruskall-Wallis non-parametric post-tests.

CD43^-/-^ septic mice exhibited a decrease in the frequency of IL-2^+^ CXCR3^+^ CD4^+^ cells relative to CD43^-/-^ sham controls (Fig 8A and 8B), suggesting a sepsis- related shift away from a Th1 phenotype. Additionally, this skewing was exaggerated in CD43^-/-^ septic mice compared to WT septic mice, where CD43^-/-^ mice exhibited a decreased frequency of IL-2^+^ CXCR3^+^ CD4^+^ cells compared to WT septic mice (Fig 8A and 8B). Furthermore, CD43^-/-^ mice also exhibited a significant increase in the frequency of IL-4^+^ CCR4^+^ CD4^+^ cells (suggestive of a Th2 polarization) compared to WT mice in both and sham and septic groups (Fig 9A and 9B). To determine whether this skewing was functionally important in the observed increase in sepsis-induced mortality in CD43-/- mice, we conducted two independent lines of investigation. First, we used systemic administration of an anti-IL-4 mAb to neutralize IL-4 in CD43-/- septic animals to determine whether this would improve mortality. Animals were treated with anti-IL-4 as described in Materials and Methods on days 0, 2, 4, and 6 post sepsis and monitored for survival. Results indicated that neutralization of IL-4 did not improve sepsis mortality in CD43-/- hosts.

Next, we queried whether adding back in vitro polarized Th1 cells into CD43-/- septic hosts would improve sepsis-induced mortality. Splenocytes from CD43-/- mice were cultured in vitro with anti-CD3/CD28 in Th1 polarizing conditions (IL-12, IL-2, and anti-IL-4 as described in Materials and Methods). In vitro polarized CD43-/- Th1 cells were adoptively transferred into CD43-/- hosts one day post-CLP. Results indicated that the reconstitution of Th1 cells into CD43-/- septic hosts did not improve sepsis mortality. Taken together, these data strongly suggest that Th2 polarization is not the mechanism underlying the increased mortality observed in CD43-/- septic animals.

### Th17 cell populations are also altered in CD43^-/-^ septic mice

**Fig 10:**
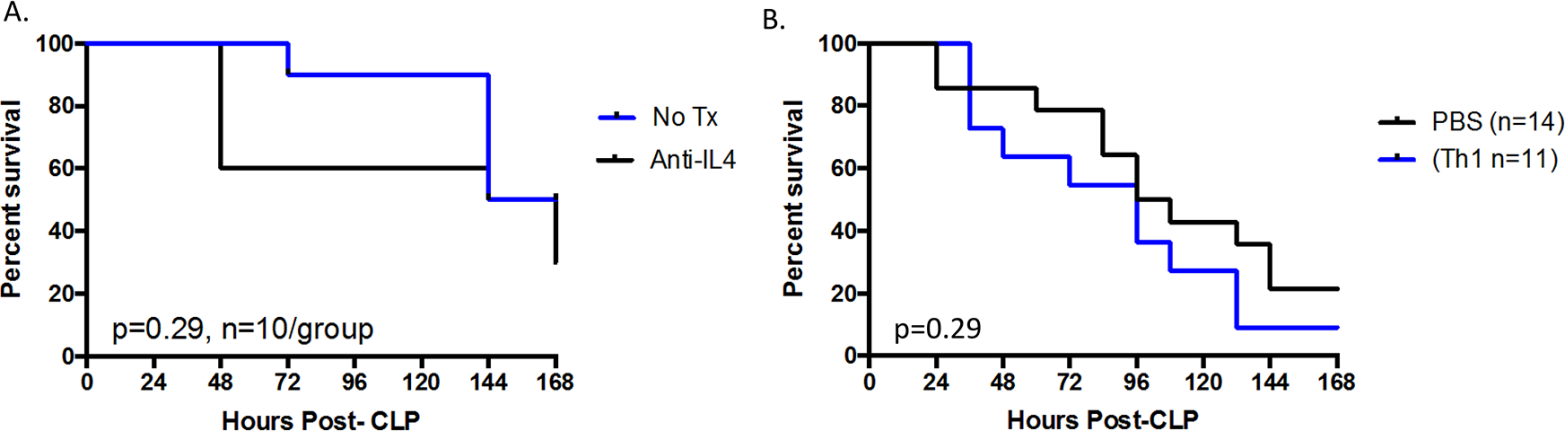
Neutralizing IL-4 or adding back Th1 cells does not increase sepsis survival in CD43^-/-^ hosts. A, B, CD43^-/-^ animals were treated with anti-IL-4 as described in Materials and Methods and followed for survival. Statistical comparisons were performed by Mann-Whitney non-parametric test. There was no difference in survival between the groups. B, In vitro-polarized Th1 cells (or vehicle alone PBS control) were adoptively transferred into septic CD43^-/-^ hosts as described in Materials and Methods and monitored for survival. Survival curves were analyzed by log-rank test.

**Fig 11:**
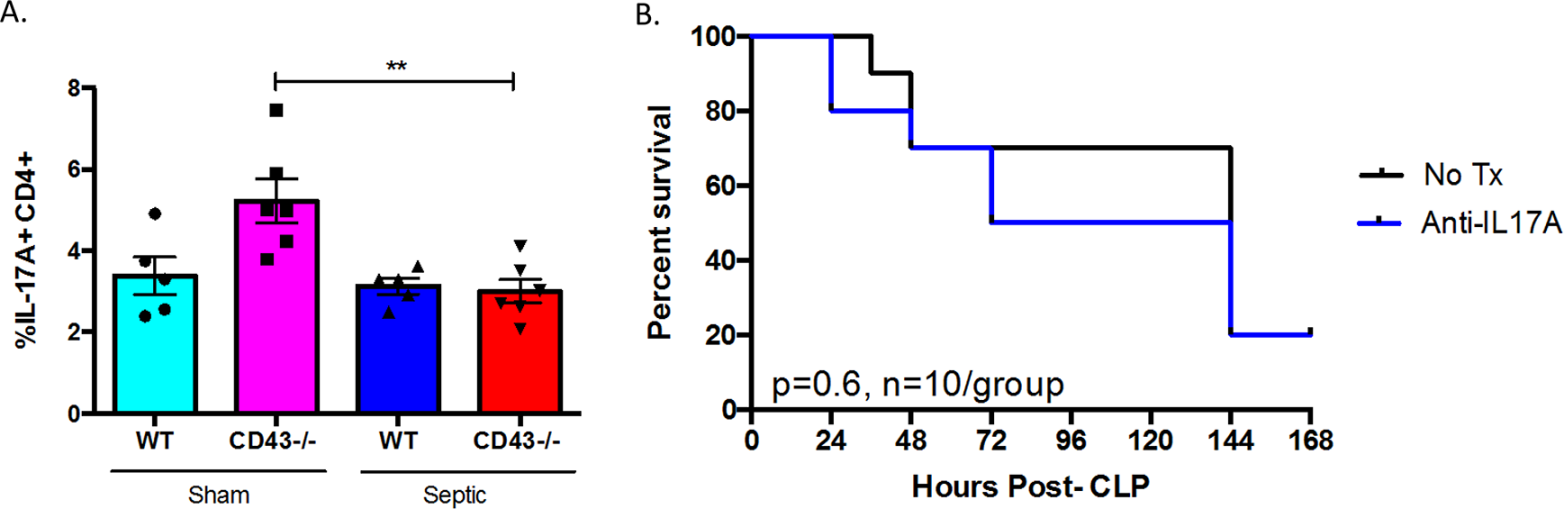
Neutralizing IL-17 does not improve sepsis survival in CD43^-/-^ hosts. A, WT and CD43^-/-^ animals were subjected to CLP and spleens were harvested at 24h. The frequency of T_H_17 cells, defined as IL-17A^+^ CD4^+^ cells, decreased in CD43^-/-^ mice after sepsis induction (5.2±0.5 vs 3±0.3, p = 0.004). Statistical comparisons were performed by Mann-Whitney non-parametric test. B, CD43^-/-^ animals were treated with anti-IL-17 as described in materials and methods and followed for survival. Survival curves were analyzed by log-rank test. There was no difference in survival between the groups.

Other helper T cell subsets are also altered in CD43^-/-^ mice after sepsis induction. Additionally, the frequency of IL-17A^+^ CD4^+^ Th17 cells significantly decreased from CD43^-/-^ sham to CD43^-/-^ septic mice, although there were no differences compared to WT controls (Fig 10B). CD43 has been shown to act as an E-selectin ligand for Th17 cells in their recruitment to peripheral inflamed tissues. Thus, to address the role of Th17 cells in sepsis-induced mortality in CD43^-/-^ hosts, we neutralized the activity of Th17 cells in CD43^-/-^ during sepsis by administering an anti-IL-17 mAb on days 0, 2, 4, and 6 post-CLP. PBS-treated CD43^-/-^ hosts were used as controls. As shown in Fig 11B, our results demonstrated that inhibiting Th17 responses via an anti-IL-17 mAb in CD43^-/-^ hosts had no impact on sepsis-induced mortality.

### Foxp3^+^ Treg are altered in CD43^-/-^ septic mice and depletion of Treg eliminates increased mortality in CD43^-/-^ septic mice

**Fig 12:**
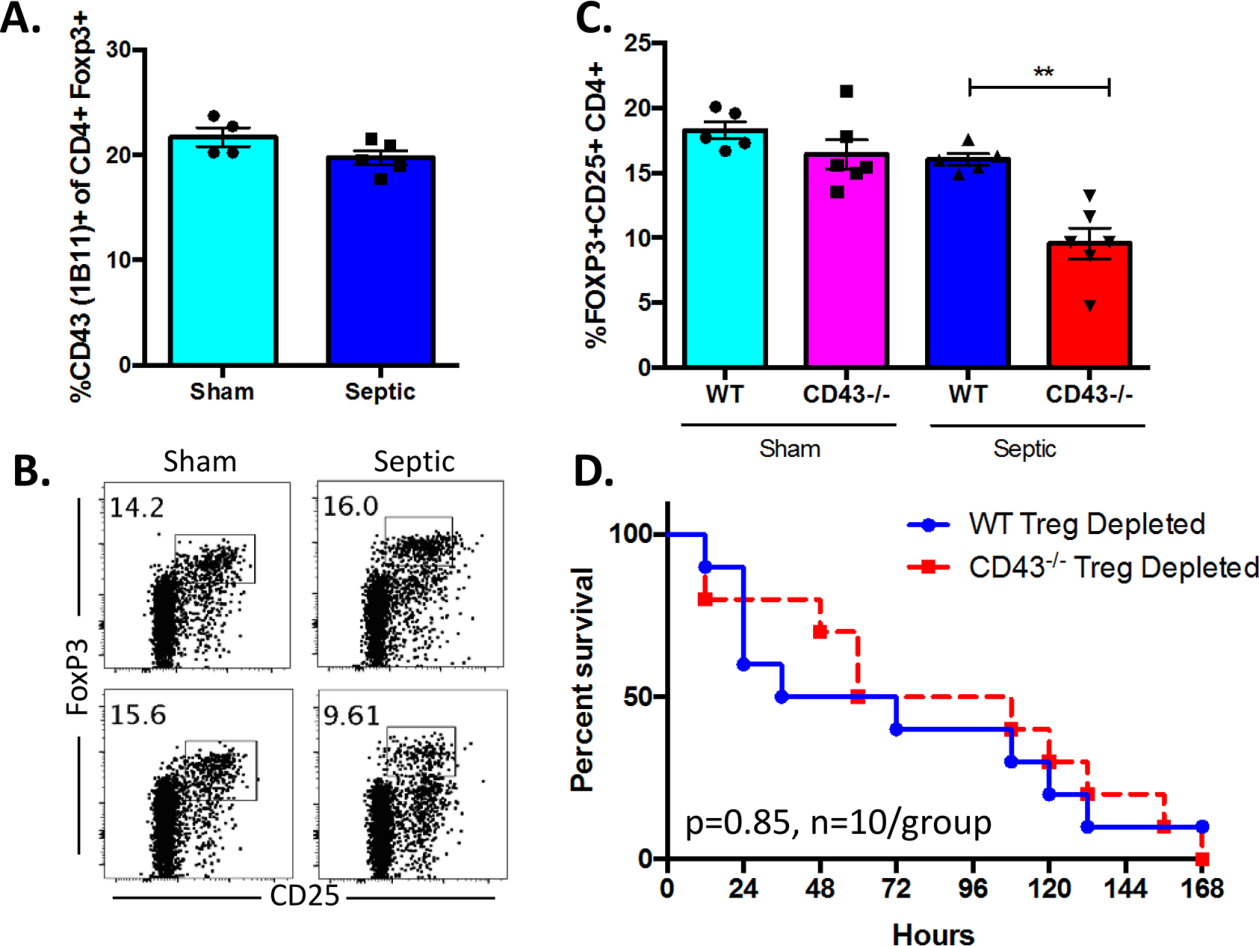
Foxp3^+^ Treg are altered in CD43^-/-^ septic mice and depletion of Treg eliminates increased mortality in CD43^-/-^ septic mice. A-C), WT and CD43^-/-^ animals were subjected to CLP and spleens were harvested at 24h. A) Expression of CD43 (1B11) on CD4^+^ Foxp3^+^ Treg was assessed in splenocytes from sham animals and or 24 h following CLP. Statistical comparisons were performed by Mann-Whitney non-parametric test. B-C) T Regulatory cells (T_Reg_ cells), defined here as Foxp3^+^ CD25^+^ CD4^+^ cells (representative flow plots shown in B), did not have an alteration in frequency between CD43^-/-^ sham and WT sham mice (16.4±1.1 vs 18.3±0.7, p>0.9); however, there was a significant reduction in %T_Reg_ cells in CD43^-/-^ septic mice compared to WT septic controls (9.6±1.2 vs 16±0.5, p = 0.004) (C). D) B6 and CD43^-/-^ animals were left untreated or treated with the Treg-depleting PC.61 mAb as described in Materials and Methods and monitored for survival. Survival curves were analyzed by log-rank test.

Finally, we assess the role of regulatory T cells (T_Reg_ cells), defined as Foxp3^+^ CD25^+^ CD4^+^ cells, in the increased mortality observed in CD43^-/-^ animals during sepsis. CD43 is expressed on Foxp3^+^ Treg and has been shown to negatively regulate Treg expansion via interaction with macrophage-expressed sialoadhesins [30]. We found that ~22% of Foxp3^+^ Treg stained positive for the 1B11 glycoform in sham animals (Fig 12A). However, this expression was not altered during sepsis (Fig 12A). Instead, overall frequencies of Foxp3^+^ CD25^+^ CD4^+^ Treg were significantly decreased in CD43^-/-^ septic mice compared to WT septic mice (Fig 12B and 12C). To determine if this decrease functionally impacts sepsis mortality in this system, we depleted CD25^+^ Treg via administration of the PC.61 mAb in both WT and CD43^-/-^ mice on days -1 and 3 relative to CLP surgery as described in Materials and Methods. Results indicated sepsis mortality was not different between WT and CD43^-/-^ mice in the absence of Treg (p = 0.85, n = 10 mice/group) (Fig 12D), demonstrating that depletion of CD25^+^ Treg eliminated the survival disadvantage that we had previously observed in CD43^-/-^ relative to WT septic hosts (Fig 2). These data suggest that the reduction in Treg in CD43^-/-^ animals during sepsis may causally underlie the observed increase in sepsis-induced mortality.

## Discussion

While previous data has highlighted the role of the transmembrane protein CD43 in a myriad of T cell functions, little is known about the role of CD43 in sepsis immunopathology. This study aimed to utilize a CD43^-/-^ model to further understand the impact of CD43 and the immunological derangements that occur in the senescent phase of sepsis. We found a significant increase in sepsis-induced mortality in CD43^-/-^ mice. This increase in mortality was associated with alterations in central memory cell apoptosis, as well as alterations within the CD4^+^ T cell compartment. While IL-2-secreting Th1-like, IL-17-secreting Th17, and CD25^+^ Foxp3^+^ Treg populations were all decreased, IL-4-secreting Th2-like cells were increased. Importantly, depleting CD25^+^ Foxp3^+^ Treg erased the observed difference in mortality between WT and CD43^-/-^ mice, while neutralizing IL-4 or adding back IFN-g-secreting Th1-like cells had no impact on sepsis survival in CD43^-/-^ hosts. Together, these data suggest that the decrease in CD25^+^ Foxp3^+^ Treg in CD43^-/-^ septic hosts may underlie the increased mortality observed in these animals during sepsis.

Although generally a marker of activation, overexpression of CD43 has been implicated in the induction of apoptosis following ligation with galectin-1[29]. Importantly, T cells expressing the core 2 *O*-glycan isoform of CD43 recognized by 1B11 have been shown to be significantly more susceptible to galectin-1-induced apoptosis as compared to T cells expressing the core 1 *O*-glycan isoform recognized by clone S7 [29]. Of note, our results indicate that expression of the CD43 isoform 1B11 significantly increases on both CD4^+^ and CD8^+^ T cells following sepsis, suggesting that this upregulation may play a role in sepsis-induced T cell apoptosis. However, not all T cell subsets exhibited reduced apoptosis in the absence of CD43: our data showed that CD44^HI^ CD62L^HI^ central memory cells (both CD4^+^s and CD8^+^s) exhibited increased apoptosis in the absence of CD43 while naïve CD4^+^s and CD8^+^s exhibited decreased apoptosis in the absence of CD43, even though CD44^HI^ subsets contained similar frequencies of the 1B11 isoform as did total T cell populations (Fig 1). Thus, further investigation into the mechanism by which CD43 impacts T cell apoptosis during sepsis is warranted.

Similar to previously published data, we have shown that CD43 is associated with a T_H_1 phenotype. Early activation of CD43 results in secretion of pro-inflammatory cytokines, cellular proliferation, and most significantly, production of high levels of IL-2 [34], which promotes T_H_1 differentiation. Reciprocally, utilization of CD43^-/-^ models have demonstrated a reduction in production of IL-2 and IFN-γ, resulting in decreased T_H_1 cell populations, as well as an increase in production of IL-4, which drives expansion of T_H_2 cells [28]. The balance of T_H_1/T_H_2 cell has been investigated extensively in inflammatory and traumatic disease. Alterations in cytokine production during critical illness, specifically suppression of IL-2 and IFN-γ, promote a predominant T_H_2 environment [20, 35, 36], resulting in enduring ineffective T cell responses. Our findings also demonstrate that CD43 drives a T_H_1 phenotype, as CD43^-/-^ demonstrate significantly decreased IL-2^+^ CXCR3^+^ CD4^+^ (T_H_1-like) cells with a concomitant increase in IL-4^+^ CCR4^+^ CD4^+^ (T_H_2-like) cells in sepsis. However, this finding of decreased IL-2-secreting / increased IL-4-secreting Th cells does not appear to be a major driver of mortality during sepsis, as either neutralizing IL-4 nor adding back IFN-g-secreting Th1-like cells impacted mortality in CD43^-/-^ mice.

Finally, our findings indicate that CD43 deficient mice exhibit a significant decrease in regulatory T cell (T_Reg_) populations during sepsis. While the role of Foxp3^+^ Treg during sepsis was initially somewhat controversial, the balance of studies published over the last decade have demonstrated that Foxp3^+^ Treg are likely beneficial during sepsis. For example, seminal studies revealed that the adoptive transfer of CD4^+^ CD25^+^ T cells increased bacterial clearance and improved survival in a CLP model [24]. Later studies using the Foxp3-diptheria toxin “DEREG” mice revealed that Foxp3^+^ Treg contribute to the recovery of animals from a septic insult [23]. These data are therefore consistent with our findings that decreased Foxp3^+^ Treg in CD43^-/-^ septic mice may mechanistically underlie the observed worsened survival. Why are Foxp3^+^ Treg populations reduced during sepsis in the absence of CD43? This question remains to be answered, as both the role of CD43 on Treg and the alterations in CD43 ligands during sepsis have been only very minimally explored. One study showed that sialoadhesin (Sn) expressed by monocytic cells can bind to CD43 expressed on Foxp3^+^ Treg and negatively regulate their expansion [30], and thus future investigation of the frequency and localization of Sn+ monocytes during sepsis may yield important insights. In sum, our data demonstrate that CD43 deficiency during sepsis results in increased mortality, that may be causally driven by a decrease in CD25^+^ Foxp3^+^ Treg populations. Further investigation into the function of CD43 on regulatory T cells during sepsis is warranted in this regard.
